# Simple screening procedure for 72 synthetic cannabinoids in whole blood by liquid chromatography–tandem mass spectrometry

**DOI:** 10.1007/s11419-017-0401-x

**Published:** 2018-01-31

**Authors:** Katarzyna Ambroziak, Piotr Adamowicz

**Affiliations:** Institute of Forensic Research, Westerplatte 9, 31-033 Kraków, Poland

**Keywords:** Synthetic cannabinoids, ‘Legal highs’, Whole-blood screening analysis, LC–MS/MS

## Abstract

**Purpose:**

In recent years, many synthetic cannabinoids (SCs) have appeared on the drug market. Despite the increasing number of SCs, there are few comprehensive screening methods for their detection in biological specimens. In this context, the purpose of this study was to develop a fast and simple liquid chromatography–tandem mass spectrometry screening procedure for detection and identification of SCs in whole blood.

**Methods:**

The elaborated qualitative screening method allows the simultaneous detection and identification of 72 compounds from different chemical groups: naphthoylindoles, naphthoylindazoles, benzoylindoles, phenylacetylindoles, tetramethylcyclopropylindoles, indole-3-carboxylic acid esters, indole-3-carboxylic acid amides, indazole-3-carboxylic acid amides, and others. Whole-blood samples (0.2 mL) were precipitated with acetonitrile (0.6 mL). The separation was achieved with the gradient of the mobile phase composition (0.1% formic acid in acetonitrile and 0.1% formic acid in water) and the gradient of the flow rate (0.5–0.8 mL/min) in 16 min. Detection of all compounds was based on dynamic multiple reaction monitoring.

**Results:**

Mass spectrometer parameters for all compounds were presented. All of the compounds were well-separated by their retention times and/or transitions. The limits of detection (LODs) for 50 compounds were in the range 0.01–0.48 ng/mL.

**Conclusions:**

Estimated LODs make this assay suitable for the analysis of biological material. The procedure can be easily expanded for more substances, which is an indispensable advantage in the dynamically developing drug market. It can have wide application in various analytical forensic and clinical laboratories.

## Introduction

One of the largest groups of new drugs that are monitored by the European Monitoring Centre for Drugs and Drug Addiction (EMCDDA) is synthetic cannabinoids (SCs). The first popular synthetic cannabinoid, JWH-018, has been available on the drug market since at least 2006, and it was identified for the first time in 2008 in the smoking mixture ‘Spice’. Since then, almost 170 SCs have been detected by the Early Warning System (EWS) of the European Union [1]. The United Nations Office on Drugs and Crime (UNODC) is another organization responsible for controlling the global drug situation. Between 2009 and 2016, over 240 SCs have been reported by 65 countries to this organization [2]. The problem is constantly increasing, because SCs that become subjected to legal control are replaced by new uncontrolled substances. Currently, around 20–30 new SCs are identified each year [1]. The popularity of this group of compounds is also confirmed by numerous seizures of SCs [1, 3].

Until recently, SCs were most often sold as plant preparations, but lately they have also appeared on the market in the forms of powders as well as liquids for use with electronic cigarettes. More recently, even tablets and capsules have started to appear on the market [2–4]. Mixtures of herbs or other types of plants (e.g., dried leaves of Damiana *Turnera diffusa* or *Lamiaceae* herbs such as *Melissa* and *Thymus*) are in reality only a base into which the SCs are applied (mostly using solvents to dissolve the chemicals first). Therefore, the term ‘herbal highs’ used for them is very misleading. The form of SC preparations makes them generally administered by smoking (usually as a joint or in a water-pipe), but sometimes they are taken orally.

SCs are chemically very diverse, and currently 14 recognisable chemical families of these substances are known [3]. However, all of them share the ability to affect the cannabinoid receptors in the body, mimicking the effects of Δ^9^-tetrahydrocannabinol (THC). The physiological and psychotropic effects of SCs are mainly associated with the cannabinoid CB_1_ receptor [2]. The *K*_i_ inhibition constant is the measure of affinity to the cannabinoid receptor; the greater the affinity, the smaller the constant. As a result, a lower *K*_i_ for a substance means that a lower dose is needed to produce the desired effect. The affinities of most SCs are smaller when compared to THC, which means that the effects of many SCs are stronger. The first identified SCs (e.g., JWH-018) were about four times stronger than the natural component of cannabis, and currently sold substances are 50–1000 times stronger. Active doses of most SCs are very small, even less than 1 mg. Such doses along with a rapid metabolism and numerous metabolic pathways results in very low observed blood concentrations.

Although SCs are advertised as ‘legal’ and ‘safe’ substitutes for marijuana, they most often do not contain cannabis and are extremely dangerous. Administration of SCs represents a huge danger to human health and life. Many unpredictable and serious adverse health effects have been reported; neurological and cardiovascular effects are most common. Agitation, aggressive behaviour, delirium, seizures, convulsions, somnolence, anxiety, hallucinations and psychoses, memory loss, nausea/vomiting, hypertension, tachycardia, myocardial infraction, acute kidney injury, loss of consciousness, and coma have been observed in intoxicated patients. Unfortunately, the administration of SCs can also lead to death, often caused by acute circulatory-respiratory failures, usually preceded by a heart attack [3–9]. SCs are the cause of some large outbreaks of intoxication [3, 5].

The number of SCs, their chemical diversity and variability on the drug market as well as low concentrations in biological specimens cause many analytical problems. The development of detection methods of these substances in biological material is therefore challenging. Tools for reliable and rapid identification of SCs in biological specimens are necessary for forensic and clinical toxicology laboratories, in order to detect them in judicial cases and to diagnose intoxications. Commercially available immunoassays are limited to a set number of drugs and always delayed for newly emerging substances. In this situation, only hyphenated techniques, especially liquid chromatography coupled with mass spectrometry (LC–MS), and in particular, combined with tandem mass spectrometry (LC–MS/MS) methods, provide the sensitivity and selectivity necessary for simultaneous screening analysis for many SCs in biological materials.

Currently, several LC–MS methods exist for the detection of SCs in biological samples, but comprehensive screening methods incorporating a high number of compounds are still very limited [10]. Relatively large groups of SCs were analysed in whole blood [11], serum [12], hair [13], and oral fluid [14–16]. These methods included up to several dozen substances. Methods of analysis of SCs in urine also covered many metabolites due to the fact that unchanged compounds are often not found in this material and main target compounds are metabolites [9].

In this context, the aim of this work was to develop a fast and simple LC–MS/MS screening procedure for detection and identification of a large group of SCs in whole blood in a single run.

## Materials and methods

### Chemicals

All certified substances used during development and validation of the method were purchased from Cayman Chemical Company (Ann Arbor, MI, USA), LGC Standards (Łomianki, Poland), Lipomed (Arlesheim, Switzerland), Cerilliant (Round Rock, TX, USA) and NMI (Australian Government National Measurement Institute, Pymble, Australia). All solvents (methanol, acetonitrile, and ethyl acetate) as well as formic acid (≥ 98%) were of HPLC grade purchased from Merck (Warsaw, Poland).

### Biological material

Drug-free (blank) blood samples used for the development and validation of the method were obtained from a regional blood donation centre. Forensic blood samples were sent to the Institute of Forensic Research, Kraków, Poland, in the first half of 2017, together with the provisions of investigative bodies to carry out toxicological analysis for the presence of psychoactive substances.

### Standards and spiked whole-blood samples

Stock and working methanolic solutions of SCs were stored below −20 °C. Selected SCs were spiked into drug-free whole-blood samples to achieve the following concentrations: 0.1, 0.2, 0.5, 1, 2, 5, 10, 20, 50, and 100 ng/mL of blood [low concentrations were used for estimation of limits of detection (LODs), intermediate levels for matrix effect experiments, while high concentrations were used for carryover experiments]. JWH-018-*d*_9_ spiking solution (1 μg/mL) was prepared for use as the internal standard (IS).

### Specimen procedure

The whole-blood samples (0.2 mL) were placed in plastic 2.0-mL vials. Next, 20 µL of 100 ng/mL methanolic solution of JWH-018-*d*_9_ (IS) was added to obtain a final concentration of 10 ng/mL of blood. The samples were precipitated with acetonitrile. For this purpose, 0.6 mL of iced acetonitrile were added dropwise. During the addition, the sample was continuously mixed on a vortex mixer. In the next step, the samples were mixed for 5 min and centrifuged at 13,000 rpm for 5 min. The organic solvent was transferred to a 2-mL glass vials and then the acetonitrile was evaporated to dryness under an air stream at 30 °C. The dry residues were dissolved in 100 µL of a mixture of 0.1% formic acid in acetonitrile/0.1% formic acid in water (1:4, v/v), and the solution was transferred to inserts for autosampler vials. The injection volume was 10 µL.

### Chromatographic and spectrometric conditions

Analyses were performed on an Agilent 1200 series liquid chromatograph connected to a 6460 Triple Quad mass spectrometer (Agilent Technologies, Santa Clara, CA, USA). The separation was achieved using a Kinetex C18 2.6u 100 Å (100 × 4.6 mm) column (Phenomenex, Torrance, CA, USA). The mobile phase consisted of a mixture of 0.1% formic acid in acetonitrile (v/v) and 0.1% formic acid in water (v/v). The variable flow rate and linear gradient conditions used are shown in Table 1. Dynamic multiple reaction monitoring (MRM) with positive ion detection was applied (retention time window was set at 1 min). The precursor ions and three (for AM-1220 and ADB-FUBINACA, two) product ions for each compound were monitored. The total number of transitions monitored was 217, and the total analytical run time was 16 min. The mass detector parameters were as follows: capillary voltage 3000 V, gas flow (nitrogen) 10 L/min, gas temperature 325 °C, sheath gas flow 11 L/min, sheath gas temperature 325 °C, nebulizer pressure 40 psi and cycle time 700 ms. The transitions, fragmentor voltages, collision energies, and retention times for each compound are presented in Table 2. The apparatus maintenance and results analyses were conducted using MassHunter software by Agilent Technologies (version B.04.01).

**Table 1:** The mobile phase gradient and flow rate conditions of the developed method

Time (min)	Mobile phase gradient		Flow rate (mL/min)
	0.1% formic acid in water (%)	0.1% formic acid in acetonitrile (%)	
0.0	60	40	0.5
1.0	60	40	0.5
3.5	40	60	0.8
4.5	10	90	0.8
10.0	10	90	0.8
10.5	60	40	0.5
16.0	60	40	0.5

The linear gradient elution at 1.0–3.5, 3.5–4.5, and 10.0–10.5 min was applied

**Table 2:** List of synthetic cannabinoids covered by the developed method with their molecular formulas, precursor and product ions, mass spectrometer parameters, retention times, and limits of detection

Compound	Molecular formula	Precursor ion	Product ions	Relative ion transition intensities	Fragmentor voltage (V)	Collision energies (V)	Retention time (min)	LOD (ng/mL)
5Cl-UR-144	C_21_H_28_ClNO	346.2	248.0	0.51 1.00 0.40	91	24 24 44	7.49	0.02
			125.1					
			55.1					
5Cl-AKB-48	C_23_H_30_ClN_3_O	400.2	77.1	0.12 0.12 1.00	108	20 56 116	8.27	–
			93.1					
			135.1					
5F-AB-PINACA	C_18_H_25_FN_4_O_2_	349.2	332.1	0.64 0.97 1.00	68	4 12 20	5.03	0.01
			304.1					
			233.1					
5F-ADB (5F-MDMB-PINACA)	C_20_H_28_FN_3_O_3_	378.2	41.2	0.23 0.46 1.00	102	48 80 60	6.75	–
			90.0					
			145.0					
5F-ADB-PINACA	C_19_H_27_FN_4_O_2_	363.2	346.2	0.72 1.00 0.97	83	0 8 20	5.43	–
			318.2					
			233.1					
5F-AKB-48 (5F-APINACA)	C_23_H_30_FN_3_O	384.2	135.1	1.00 0.15 0.15	99	20 56 60	7.74	0.01
			93.0					
			79.1					
5F-AMB (5F-MMB-PINACA)	C_19_H_26_FN_3_O_3_	364.2	304.1	0.74 1.00 0.71	91	12 20 44	6.58	0.02
			233.1					
			145.0					
5F-APP-PINACA (PX-2)	C_22_H_25_FN_4_O_2_	397.2	145.0	0.56 1.00 0.60	84	4 8 52	5.75	–
			352.2					
			380.2					
5F-MN-18	C_23_H_22_FN_3_O	376.2	90.0	0.24 1.00 0.51	102	12 40 76	7.11	–
			233.1					
			145.0					
5F-NNEI	C_24_H_23_FN_2_O	375.2	232.3	1.00 0.50 0.20	99	20 44 60	6.63	0.06
			144.1					
			115.8					
5F-NPB-22	C_22_H_20_FN_3_O_2_	378.2	233.1	1.00 0.42 0.75	101	12 20 40	6.43	–
			213.1					
			145.1					
5F-PB-22	C_23_H_21_FN_2_O_2_	377.2	232.1	1.00 0.48 0.21	43	8 40 60	6.62	0.01
			144.0					
			116.0					
5F-PY-PICA	C_18_H_23_FN_2_O	303.2	55.2	1.00 0.20 0.64	118	36 76 36	5.80	–
			89.1					
			144.0					
5F-SDB-005	C_23_H_21_FN_2_O_2_	377.2	145.0	0.50 1.00 0.22	84	8 40 76	7.07	–
			233.1					
			90.0					
5F-THJ	C_22_H_21_FN_4_O	377.2	233.3	1.00 0.56 0.22	85	20 44 56	7.39	0.05
			145.1					
			41.1					
AB-005	C_23_H_32_N_2_O	353.3	125.1	0.49 0.96 1.00	43	16 20 32	3.66	0.02
			112.1					
			98.1					
AB-CHMINACA	C_20_H_28_N_4_O_2_	357.2	340.2	0.61 0.81 1.00	43	4 12 24	6.32	0.04
			312.2					
			241.1					
AB-FUBINACA	C_20_H_21_FN_4_O_2_	369.2	324.1	1.00 0.89 0.91	43	8 20 48	5.38	0.01
			253.1					
			109.0					
AB-PINACA	C_18_H_26_N_4_O_2_	331.2	314.1	0.49 0.82 1.00	62	4 8 20	5.93	0.01
			286.2					
			215.1					
AB-PINACA *N*-(2-fluorpentyl) izomer	C_18_H_25_FN_4_O_2_	349.2	233.1	1.00 0.84 0.50	86	4 8 20	5.48	–
			304.2					
			332.2					
ADAMANTYL-THPINACA (AD-THPINACA)	C_24_H_31_N_3_O_2_	394.2	77.1	0.13 0.11 1.00	108	24 60 100	7.49	–
			93.1					
			135.1					
ADB-CHMICA (MAB-CHMICA)	C_22_H_31_N_3_O_2_	370.2	353.3	0.66 1.00 0.36	93	4 20 40	6.28	–
			240.2					
			144.1					
ADB-FUBINACA	C_21_H_23_FN_4_O_2_	383.2	108.9	1.00 0.96	62	24 52	5.88	0.01
			253.0					
ADBICA	C_20_H_29_N_3_O_2_	344.2	144.0	0.50 1.00 0.76	84	4 20 44	6.14	–
			214.1					
			327.1					
AKB-48 (APINACA)	C_23_H_31_N_3_O	366.3	135.1	1.00 0.12 0.12	87	20 56 60	9.15	0.07
			93.1					
			79.1					
AM-679	C_20_H_20_INO	418.1	230.8	1.00 0.43 0.47	58	28 52 60	7.18	0.02
			203.0					
			76.0					
AM-694	C_20_H_19_FINO	436.1	230.9	1.00 0.48 0.38	141	24 48 60	6.73	0.02
			202.8					
			76.0					
AM-1220	C_26_H_26_N_2_O	383.2	112.1	1.00 0.78	114	16 32	2.96	–
			98.1					
AM-1248	C_26_H_34_N_2_O	391.3	135.2	1.00 0.35 0.28	70	36 36 56	4.47	0.02
			112.2					
			98.3					
AM-2201	C_24_H_22_FNO	360.2	127.0	0.88 1.00 0.14	174	24 24 52	6.93	0.01
			155.0					
			232.0					
AM-2233	C_22_H_23_IN_2_O	459.1	112.1	0.61 1.00 0.19	159	20 28 60	2.53	0.01
			98.1					
			70.1					
AMB-CHMICA	C_22_H_30_N_2_O_3_	371.2	240.2	1.00 0.34 0.26	83	12 36 52	6.89	–
			144.1					
			55.2					
APICA	C_10_H_12_NO_5_P	365.0	135.1	1.00 0.25 0.23	180	28 48 56	7.83	0.06
			93.0					
			79.1					
BB-22 (QUCHIC)	C_25_H_24_N_2_O_2_	385.2	240.3	1.00 0.53 0.46	58	24 40 60	7.38	0.12
			144.1					
			55.1					
EAM-2201	C_26_H_26_FNO	388.2	232.1	0.56 1.00 0.39	91	24 24 44	7.25	0.48
			183.1					
			155.1					
EG-018	C_28_H_25_NO	392.2	264.2	0.13 1.00 0.90	128	24 24 52	8.82	0.03
			155.1					
			127.1					
EMB-FUBINACA (AEB-FUBINACA)	C_22_H_24_FN_3_O_3_	398.2	83.1	0.27 1.00 0.73	82	12 44 108	6.88	–
			109.0					
			324.2					
JWH-007	C_25_H_25_NO	356.2	288.1	0.13 1.00 0.95	141	20 20 52	7.63	0.01
			155.0					
			127.0					
JWH-015	C_23_H_21_NO	328.2	200.0	0.11 1.00 0.81	130	20 20 44	7.06	0.01
			155.0					
			127.0					
JWH-018 (AM-678)	C_24_H_23_NO	342.2	214.1	0.16 0.98 1.00	130	16 20 48	7.49	0.02
			154.9					
			127.0					
JWH-018-*d*_9_ (internal standard)	C_24_H_14_D_9_NO	351.2	223.1	0.17 1.00 0.99	118	20 24 52	7.47	–
			155.0					
			127.0					
JWH-019	C_25_H_25_NO	356.2	228.0	0.10 0.93 1.00	145	20 24 48	7.82	0.01
			155.0					
			127.0					
JWH-073	C_23_H_21_NO	328.2	200.1	0.12 1.00 0.87	130	16 20 44	7.22	–
			155.0					
			127.0					
JWH-081	C_25_H_25_NO_2_	372.2	214.1	0.28 1.00 0.45	182	20 20 44	7.61	0.01
			185.0					
			157.0					
JWH-098	C_26_H_27_NO_2_	386.2	185.0	1.00 0.38 0.24	155	24 44 60	7.75	0.03
			157.0					
			127.0					
JWH-122	C_25_H_25_NO	356.2	169.0	1.00 0.65 0.51	184	24 48 60	7.77	0.01
			141.0					
			115.0					
JWH-182	C_27_H_29_NO	384.2	214.1	0.34 1.00 0.79	176	24 24 48	8.49	0.01
			197.1					
			141.0					
JWH-200	C_25_H_24_N_2_O_2_	385.2	155.0	1.00 0.52 0.46	128	16 48 24	2.91	0.01
			127.0					
			114.1					
JWH-201	C_22_H_25_NO_2_	336.2	134.9	0.48 1.00 0.47	99	28 28 60	6.87	0.01
			120.8					
			77.1					
JWH-203	C_21_H_22_ClNO	340.2	214.1	0.12 0.10 1.00	184	24 16 28	7.31	0.01
			188.1					
			125.0					
JWH-210	C_26_H_27_NO	370.2	214.1	0.33 1.00 0.35	114	20 24 52	8.08	0.01
			183.1					
			153.1					
JWH-250	C_22_H_25_NO_2_	336.2	144.0	0.11 1.00 0.45	128	32 16 44	7.12	0.01
			121.0					
			91.1					
JWH-251	C_22_H_25_NO	320.2	214.1	0.60 0.45 1.00	126	20 32 20	7.29	0.01
			144.0					
			105.0					
JWH-302	C_22_H_25_NO_2_	336.2	213.9	0.50 0.50 1.00	70	24 44 20	7.02	0.01
			143.9					
			121.1					
JWH-307	C_26_H_24_FNO	386.2	155.0	1.00 0.87 0.03	147	20 56 60	7.69	0.07
			127.0					
			77.1					
JWH-387	C_24_H_22_BrNO	420.1	232.9	1.00 0.53 0.90	64	28 52 60	8.13	–
			204.9					
			126.1					
JWH-398	C_24_H_22_ClNO	378.2	191.0	1.00 0.92 0.54	151	20 44 60	8.10	0.03
			163.0					
			126.1					
JWH-412	C_24_H_22_FNO	360.2	173.0	1.00 0.57 0.23	85	24 56 60	7.67	0.05
			145.0					
			125.0					
MAB-CHMINACA (ADB-CHMINACA)	C_21_H_30_N_4_O_2_	371.2	354.2	0.61 0.88 1.00	43	4 12 24	6.65	0.01
			326.2					
			241.1					
MA-CHMINACA (AMB-CHMINACA)	C_21_H_29_N_3_O_3_	372.2	312.3	0.63 1.00 0.40	95	8 20 36	7.34	–
			145.1					
			55.2					
MDMB-CHMICA (MMB-CHMINACA)	C_23_H_32_N_2_O_3_	385.2	240.1	1.00 0.39 0.25	41	12 36 60	7.23	0.01
			144.0					
			55.1					
MDMB-FUBINACA (FUB-MDMB)	C_22_H_24_FN_3_O_3_	398.2	338.2	0.75 1.00 0.93	97	8 20 44	6.83	–
			253.1					
			109.1					
MMB-2201 (AMB-PICA)	C_20_H_27_FN_2_O_3_	363.2	232.2	1.00 0.41 0.19	99	8 36 60	6.18	–
			144.1					
			116.1					
MMB-FUBINACA (AMB-FUBINACA)	C_21_H_22_FN_3_O_3_	384.2	324.1	0.57 1.00 0.19	18	8 40 100	6.60	–
			109.0					
			83.0					
MN-018	C_23_H_23_N_3_O	358.2	215.1	1.00 0.69 0.20	102	12 36 72	7.86	–
			145.0					
			90.1					
PB-22 (QUPIC)	C_23_H_22_N_2_O_2_	359.2	214.2	1.00 0.37 0.17	62	8 40 48	7.09	0.01
			144.1					
			43.2					
RCS-4	C_21_H_23_NO_2_	322.2	135.0	1.00 0.10 0.26	188	20 60 56	7.05	0.01
			92.0					
			77.1					
STS-135	C_24_H_31_FN_2_O	383.2	135.1	1.00 0.19 0.18	170	32 56 60	7.17	0.01
			93.1					
			79.1					
THJ-018	C_23_H_22_N_2_O	343.2	215.1	1.00 0.71 0.30	89	12 36 60	7.80	0.04
			145.0					
			90.0					
THJ-2201	C_23_H_21_FN_2_O	361.2	233.1	1.00 0.40 0.61	114	12 24 36	7.15	0.01
			213.1					
			145.0					
UR-144	C_21_H_29_NO	312.2	214.1	0.33 1.00 0.43	112	20 20 40	7.94	0.24
			125.0					
			55.1					
URB-597	C_20_H_22_N_2_O_3_	339.2	214.0	1.00 0.80 0.50	143	4 16 40	5.55	0.01
			197.0					
			153.0					
XLR-11 (5-FUR-144)	C_21_H_28_FNO	330.2	232.0	0.33 1.00 0.36	145	24 20 40	7.27	0.30
			125.0					
			55.1					

*LOD* limit of detection

### Validation

The validation was performed for 50 compounds according to Scientific Working Group for Forensic Toxicology (SWGTOX) recommendations for screening/qualitative methods and included estimation of LODs, interference, stability and carryover studies, as well as matrix effect [17]. The LODs were estimated with the signal-to-noise (S/N) ratio equalling 3 (S/N = 3) for the transition with the lowest intensity and were calculated using MassHunter software (Agilent Technologies; peak-to-peak noise definition). The method selectivity was assessed by analyses of drug-free blood samples collected from ten persons, as well as blood samples from real expert studies, in which other substances were revealed. The carryover was assessed by the analysis of the blank blood after injection of the highest spiked blood samples (100 ng/mL). The stability was checked by the analysis of the previously prepared samples every 24 h for 3 consecutive days. Matrix effect was examined at concentrations of 1 and 10 ng/mL (*n* = 5 for each level). Matrix effects were calculated by comparing the peak areas from neat solutions of an SC (set *A*) with those from blood spiked with the same SC after extraction (set *B*). The following formula (according to Matuszewski et al. [18]) was used for calculations: matrix effect (%) = *B*/*A* × 100.

## Results

**Fig. 1:**
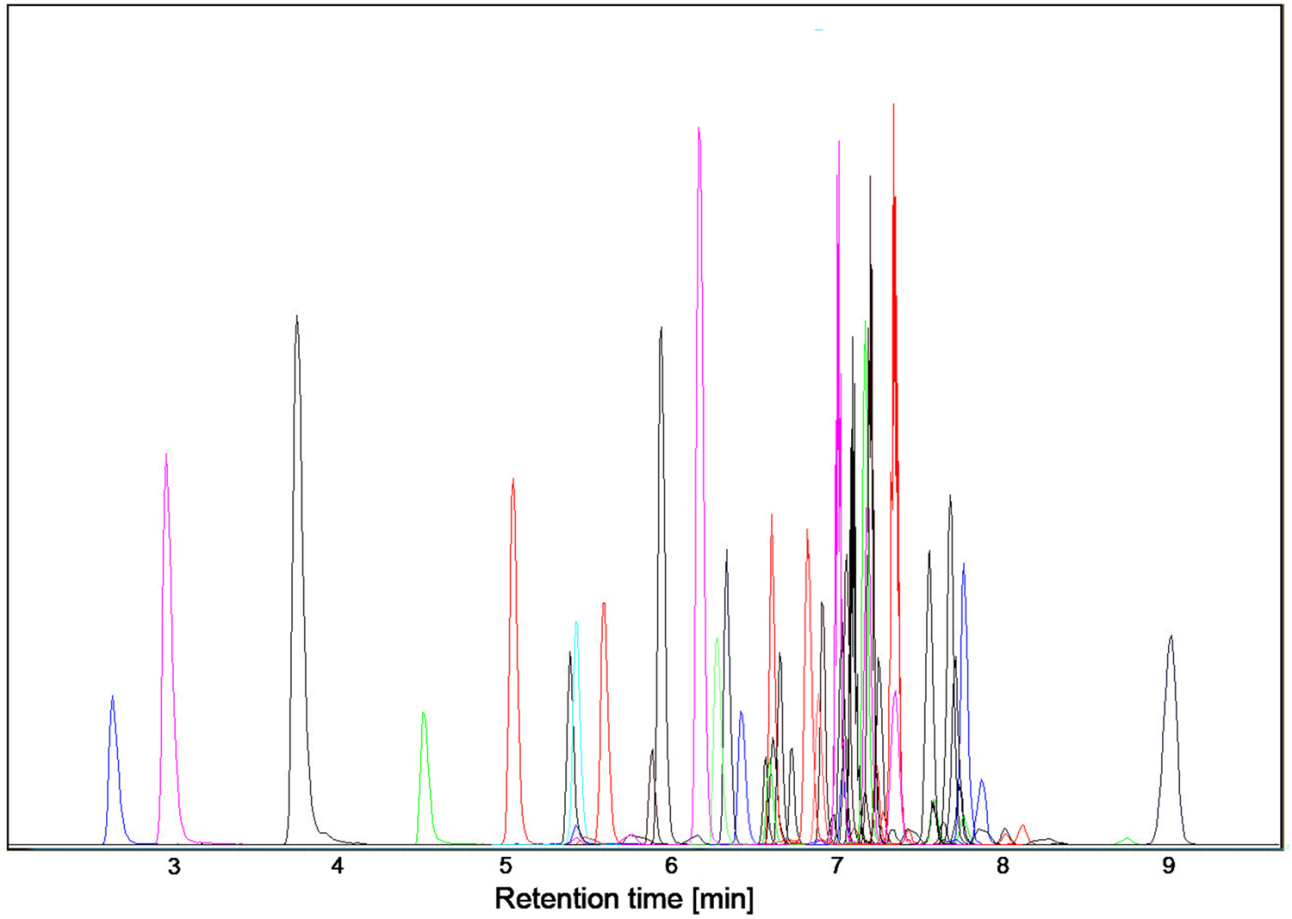
Combined multiple reaction monitoring chromatogram of the extract of whole blood spiked with analysed compounds at the concentrations of 10 ng/mL each

A screening method was developed for selective detection of 72 SCs in whole blood. The list of compounds in alphabetical order is presented in Table 2. The Agilent MassHunter Optimizer Triple Quad (version B.04.01) was used for the identification of the three most intense transitions (as well as optimum fragmentor voltages and collision energies for each transition). The dynamic MRM mode used provided an increased sensitivity, because MRM transitions were monitored only in specific detection windows that were defined ± 0.5 min from the expected retention time. The method included 217 transitions and the maximum number of concurrent ions was 117. The applied conditions allowed the separation of all the analytes in a 16-min run time. The retention times of compounds were from 2.53 to 9.15 min. Precursor and product ions, relative ion transition intensities, mass spectrometry parameters, and retention times of analysed SCs are presented in Table 2. The combined MRM chromatogram of the extracts of blood spiked with analysed compounds is shown in Fig. 1.

A validation was performed for 50 compounds to check if the method was suitable for detecting SCs in whole blood. The method was found to be selective for tested compounds; no interfering peaks were observed in the drug-free whole-blood samples taken from ten persons as well as blood samples from real expert studies (in which other substances, including psychotropic compounds, were present). The LODs values were from 0.01 to 0.48 ng/mL, depending on the compound (Table 2). The matrix effect values were from 38.9% (at a level of 1 ng/mL) and 38.5% (at a level of 10 ng/mL) to unrealistic values reaching several thousand percent (the values > 1000% were obtained for 7 of the analysed compounds). It should be noted that values less than 100% (signal suppression) were observed for only 8 analytes, and for most of compounds, they were higher than 100% (signal enhancement). The reconstituted extracts were stable for a period of more than 24 h at room temperature and 3 days at +4 °C. No carryover was observed for the analytes.

The developed qualitative screening method was applied to authentic whole-blood samples from forensic cases analysed in the Institute of Forensic Research in the first half of 2017. SCs were detected in 14 cases related to driving under the influence of drugs, road accidents, drugs possession, or drug use, as well as cases of intoxication and unintentional deaths. The following SCs were identified (the number of cases are presented in parentheses): MDMB-CHMICA (8), MDMB-CHMICA along with 5F-AMB (1), ADB-FUBINACA (3), AB-CHMINACA (1), and MAB-CHMINACA (1). The blood samples from abovementioned cases were then analysed by other quantitative methods and the determined concentrations for MDMB-CHMICA were in the range of 0.5–2.8 ng/mL, for ADB-FUBINACA in the range of 0.8–7.0 ng/mL, and for the 5F-AMB 0.7 ng/mL, AB-CHMINACA 3.5 ng/mL, and MAB-CHMINACA 3.4 ng/mL.

## Discussion

The still developing and rapidly growing drug market implies the need to develop new and sophisticated methods of identification of drugs in biological matrices. Recently, new psychotropic substances and especially SCs have been important targets of interest for both forensic and clinical laboratories. There are also more and more reports of poisonings with SCs, analytical confirmation of which is essential for professionals. The ever-increasing numbers, variability of chemical groups, many structural modifications, and low concentrations in biological materials as well as numerous and fast metabolic pathways of SCs hinder the complex analysis. Thus, the screening methods should be sensitive and discriminate a large number of different (but sometimes also with a very similar structure) substances. One of the better solutions to this problem is the use of the LC–MS/MS technique as a screening method that can be easily and quickly modified and expanded for new compounds. To be fully adequate for routine laboratory work, new procedures must also be characterised by short sample preparation and analysis. Unfortunately, there is a lack of comprehensive screening methods for simultaneous detection of a large group of SCs.

Taking into account the above issues, an attempt was made to develop an LC–MS/MS screening approach for the detection and identification of SCs in whole blood. The developed procedure allowed the simultaneous qualitative screening, detection, and identification of 72 compounds from different chemical families in whole blood: naphthoylindoles, naphthoylindazoles, benzoylindoles, phenylacetylindoles, tetramethylcyclopropylindoles, indole-3-carboxylic acid esters, indole-3-carboxylic acid amides, indazole-3-carboxylic acid amides, and others.

Coverage of many compounds with different physicochemical properties by one preparation procedure is a difficult task. Most researchers have used liquid-liquid extraction (LLE) for SCs isolation from whole blood, serum, and plasma [9]. SCs were also isolated from blood using solid-phase extraction [19]. Urine preparation included simple dilution, protein precipitation, LLE, supported liquid extraction, or salting-out LLE, and required also a hydrolysis step often with β-glucuronidase [9]. The necessity for the high-throughput and universal method of the isolation of analytes from whole blood compelled us to propose simple protein precipitation. It provided sufficient extraction efficiency, together with simplifying the extraction and significantly shortening the total time of sample preparation, which is very important in screening methods to be used for routine laboratory work. It should be emphasized that high lipophilicity of SCs facilitates their isolation from aqueous medium. For precipitation, we have used frozen acetonitrile that is one of the most efficient protein precipitants [20]. The use of ice-cold solvent improved the isolation process, which is a well-known phenomenon [21, 22].

MRM was applied in the developed method. This is a very sensitive and selective detection mode, but as the number of monitored analytes increases, the performance of the mass spectrometer decreases. The total 217 pairs of MRM were monitored in the method; for each analyte, three pairs with the exception of ADB-FUBINACA and AM-1220, for which only two transitions were observed (the fragmentation of these compounds was poor and only two transitions of high intensity were obtained). It must be noted that, for identification, it is sufficient to monitor two pairs of MRM, while in our method for most compounds, we monitored one precursor and three product ions for increased specificity. The application of dynamic MRM mode increased sensitivity by utilising the retention time window (± 0.5 min) of each analyte.

The core-shell particles in the used Kinetex C18 column provided very good resolution and high sensitivity and ensured the stability of retention times. The chromatographic separation and detection were significantly better when compared to columns with porous particles. The use of the gradient of the mobile-phase composition and the application of variable flow conditions shortened the analysis time, improved the separation of analysed compounds, and made all of the compounds well-separated by their retention times and/or transitions. These conditions allowed the chromatographic analysis and the stabilization of the phase composition in only 16 min. Most of the tested compounds were eluted in less than 7 min, in times from 2.53 to 9.15 min (Fig. 1).

The validation parameters were determined according to the recommendations of the SWGTOX. The validation was performed for 50 out of 72 compounds due to fact that the method initially covered 50 compounds, but during several months of development, a further 22 compounds were included without validation. Detection limits ranged from 0.01 to 0.48 ng/mL. It is worth emphasizing that LODs were calculated for the transition with the lowest intensity of the three monitored, and the peaks for the most intense transition can be observed even at significantly lower concentrations. LODs in other previously published methods involving extraction of a large group of SCs from blood/serum/plasma were in the range of 0.01–2.0 ng/mL [9, 23]. Comparing estimated LODs with the limits determined by the authors of other methods for SCs, it can be concluded that they were in similar ranges. Such values of LODs also demonstrated that the method was suitable for detection of SCs in authentic whole-blood samples, because the expected concentrations of these compounds in the blood specimens are low. Maximum concentrations of SCs in blood are reached quickly; drugs are metabolized smoothly and the concentrations decrease rapidly. Gurney et al. [24] described over 60 cases of SCs where the concentrations were in the range of approximately 0.1 up to 230 ng/mL. The concentrations of SCs reported in serum specimens were in the range of 0.1–190 ng/mL in poisoning cases [6]. In fatal cases, the concentrations were similarly in the range of 0.1–199 ng/mL. However, the concentrations of SCs in blood in non-fatal cases, e.g., driving under the influence of SCs, were significantly lower, and have rarely exceeded several nanograms per millilitre [5, 25, 26].

The matrix effect was also determined and the most astonishing results were those obtained for some compounds reaching a few thousand percent. We have the possible explanation for such results. Many lipids are present in whole blood, and cannabinoids are lipophilic [27, 28]. Cannabinoids are adsorbed on plastic and glass vials, meaning that during matrix effect experiments, SCs are lost in vials without matrix, while they are kept in vials with matrix due to binding with lipids [29, 30]. Such processes can greatly affect and falsify the results, and significantly increase the matrix effect values. Therefore, special attention should be paid when conducting matrix effect experiments according to the Matuszewski method, especially for compounds from the SCs group [18]. In subsequent studies, we intend to confirm (or disprove) our thesis. At this stage, the obtained results of the matrix effect appeared to be unreliable. It is also worth noting that for different blood samples (both antemortem and postmortem), no significant variation was observed for matrix effects for several tested compounds, which means that even such results are not a significant disadvantage of the developed method, but rather an advantage in view of detection sensitivity.

The developed procedure allows performing rapid qualitative screening analysis for SCs. It is being used in our laboratory in routine work for analyses of authentic blood samples collected from drug abusers, drivers, and other individuals in cases where there was a need to prove or exclude the presence of SCs. MDMB-CHMICA, ADB-FUBINACA, 5F-AMB, AB-CHMINACA, and MAB-CHMINACA were detected in 14 cases. These results correspond to the most popular SCs currently present in the Polish drug market. The application to real samples has confirmed the suitability of the proposed procedure for the toxicological screening of SCs in biological samples.

Upon comparing the developed procedure with a few published methods for SCs determination, it can be concluded that it covers a large group of compounds. Kneisel et al. [12, 14] presented LC–MS/MS methods for the analysis of 30 SCs in serum and oral fluid. Hess et al. [10] described simultaneous detection of 93 SCs in plasma using LC–MS/MS technique; however, there is a lack of screening procedures for identification of SCs in whole blood and there are great needs to analyse haemolysed whole-blood samples in forensic toxicological laboratories. The method described by Tynon et al. [11] is capable of determining 34 SCs in whole blood. Our procedure was intended for detecting 72 SCs in whole blood. The number of compounds is somewhat less than in the method developed by Hess et al. [10], but both of these methods seem complementary. As many as 39 compounds in our procedure were not covered by the Hess et al. method (many of them belong to JWH and AM SCs). Both methods are designed to analyse different materials, plasma and whole blood, and different procedures for SCs isolation (LLE and protein precipitation) were adopted. In addition, different instruments were also used for the analyses. Hess et al. [10] used a sensitive chromatograph coupled to a quadrupole linear ion trap mass spectrometer, while we applied a popular triple-quadrupole MS detector. Therefore, the parameters presented in Table 2 will be useful in laboratories using such instruments. The most important advantage of our method is that the procedure can be easily modified for more SCs. The LC–MS/MS equipment is becoming more and more popular, which makes the method more easily adaptable in other laboratories.

## Conclusions

The developed procedure enables the detection and identification of 72 SCs in blood by LC–MS/MS in a very short time. Such number of compounds is greater than in most previously published methods for the analysis of SCs in whole blood. Moreover, it is open, which provides an easy way to add new substances to the procedure. The possibility of rapidly expanding the procedure with new compounds is an indispensable advantage in the dynamically developing drug market. The method is rapid and sensitive, and the preparation procedure is not complicated and requires only 0.2 mL of whole blood. Comparing the sample preparation steps with others, it can also be assumed that it is faster, while maintaining sufficient detection limits. These aspects make it suitable for routine work in various analytical forensic and clinical laboratories. The procedure was used for the analysis of authentic samples from forensic cases confirming its suitability for this application.
